# Protective role of mouse mast cell tryptase Mcpt6 in melanoma

**DOI:** 10.1111/pcmr.12859

**Published:** 2020-01-19

**Authors:** Mirjana Grujic, Lars Hellman, Ann‐Marie Gustafson, Srinivas Akula, Fabio Rabelo Melo, Gunnar Pejler

**Affiliations:** ^1^ Department of Medical Biochemistry and Microbiology Uppsala University Uppsala Sweden; ^2^ Department of Cell and Molecular Biology Uppsala University Uppsala Sweden; ^3^ Department of Anatomy, Physiology and Biochemistry Swedish University of Agricultural Sciences Uppsala Sweden

**Keywords:** CXCL9, mast cells, Mcpt6, melanoma, tryptase

## Abstract

Tryptase‐positive mast cells populate melanomas, but it is not known whether tryptase impacts on melanoma progression. Here we addressed this and show that melanoma growth is significantly higher in tryptase‐deficient (Mcpt6^−/−^) versus wild‐type mice. Histochemical analysis showed that mast cells were frequent in the tumor stroma of both wild‐type and Mcpt6^−/−^ mice, and also revealed their presence within the tumor parenchyma. Confocal microscopy analysis revealed that tryptase was taken up by the tumor cells. Further, tryptase‐positive granules were released from mast cells and were widely distributed within the tumor tissue, suggesting that tryptase could impact on the tumor microenvironment. Indeed, gene expression analysis showed that the absence of Mcpt6 caused decreased expression of numerous genes, including *Cxcl9*, *Tgtp2,* and *Gbp10*, while the expression of *5p‐miR3098* was enhanced. The levels of CXCL9 were lower in serum from Mcpt6^−/−^ versus wild‐type mice. In further support of a functional impact of tryptase on melanoma, recombinant tryptase (Mcpt6) was taken up by cultured melanoma cells and caused reduced proliferation. Altogether, our results indicate a protective role of mast cell tryptase in melanoma growth.

**Significance**

Mast cells are known to be present in melanoma, but their role in tumor progression has not been clarified previously. Here we show that one of the enzymes expressed in large amounts by mast cells, tryptase, has a protective role in melanoma progression in a mouse model. Thereby, it is conceivable that modulation of tryptase function can represent a novel principle for interfering with melanoma growth.

## 1 INTRODUCTION

Mast cells (MCs) are multifaceted cells of the immune system. They originate in the bone marrow and circulate as immature progenitors, after which they home into tissues where they mature under the influence of local growth factors such as stem cell factor and IL‐3 (Gurish & Austen, 2012). MCs are found as resident cells of most tissues of the body, albeit particularly abundant at sites close to the exterior, such as skin and mucosal surfaces of the lung and gut. A hallmark feature of MCs is their large content of secretory granules, which are densely packed with a variety of preformed mediators. These include histamine and other biogenic amines, serglycin proteoglycans, growth factors, cytokines, lysosomal hydrolases as well as a number of MC‐restricted proteases (“MC proteases”), the latter encompassing tryptases, chymases, and carboxypeptidase A3 (CPA3) (Pejler, Rönnberg, Waern, & Wernersson, 2010; Wernersson & Pejler, 2014).

MCs can be activated in response to a variety of stimuli, including antigen‐induced cross‐linking of IgE molecules bound to their high affinity receptor on the MC surface. In addition, MC activation can be accomplished by a wide array of other stimuli such as anaphylatoxins, engagement of pattern recognition receptors, and in response to various ligands to the MRGPRX2 receptor (McNeil et al., 2015). When MCs are activated, they can respond by degranulation, whereby the preformed contents of their granules are released. MC activation can also drive the synthesis of a number of additional compounds, such as prostaglandins and leukotrienes, as well as numerous cytokines, chemokines, and growth factors (Galli, Nakae, & Tsai, 2005).

MCs are undoubtedly mostly known for their detrimental impact in allergic conditions. However, they are also known to contribute in a variety of additional disorders. In particular, there is a vast documentation supporting an involvement of MCs in various malignancies (Marichal, Tsai, & Galli, 2013; Oldford & Marshall, 2015; Ribatti & Crivellato, 2009; Varricchi et al., 2017). In this respect, a large number of clinical studies have revealed that MCs show a strong tendency to accumulate at malignant lesions. Typically, MCs are most frequently found in the tumor stroma but they can also be found within the tumor parenchyma. Clinical studies have also examined the correlation between MC presence and clinical outcome, and these studies have often led to the conclusion that MC density is associated with a poor outcome (Marichal et al., 2013; Oldford & Marshall, 2015; Ribatti & Crivellato, 2009; Varricchi et al., 2017). However, there are also numerous occasions in which MC presence appears to associate with good outcome (Marichal et al., 2013; Oldford & Marshall, 2015; Ribatti & Crivellato, 2009; Varricchi et al., 2017). Altogether, there is thus some controversy with regard to how MCs impact on cancer.

In melanoma, a number of clinical studies have revealed a strong accumulation of MCs (Ribatti, Ennas, et al., 2003; Ribatti, Vacca, et al., 2003; Siiskonen et al., 2015; Toth‐Jakatics, Jimi, Takebayashi, & Kawamoto, 2000). To gain more mechanistic insight into the role of MCs in melanoma, we previously examined melanoma progression in wild‐type (WT) mice versus mice that lacked connective tissue‐type MCs altogether (Dudeck et al., 2011), using a model of melanoma colonization to the lung. In that study, we found that MCs had an overall detrimental impact (Öhrvik et al., 2016) and to dissect the underlying mechanism we assessed mice lacking all of the MC proteases in the same model. Strikingly, we found that mice with global MC protease deficiency, contrary to the mice lacking MCs altogether, developed larger tumors than did WT mice (Grujic et al., 2017). This suggests that the combined action of the MC proteases serves a protective function in this model of melanoma, that is, despite the overall negative impact of MCs. Further, the data indicated that the protective function of the MC proteases could be related to CXCL16/CD1d‐dependent effects on iNKT cell populations (Grujic et al., 2017).

Collectively, these findings indicate that MCs harbor both detrimental and protective activities with respect to tumor progression, and the overall impact of MCs may thus reflect the balance between such activities.

The aim of this study was to further dissect the impact of the MC proteases on melanoma progression. We thereby focused on tryptase, based on our previous observation that tryptase in certain settings can have an anti‐proliferative action (Melo et al., 2017). For this purpose, we evaluated mice lacking tryptase Mcpt6 in a subcutaneous model of melanoma. Our findings reveal a protective function of tryptase in melanoma progression, hence shedding further light into the role of MCs in tumor development.

## 2 MATERIALS AND METHODS

### 2.1 Mice

WT and Mcpt6‐deficient mice were all on C57BL/6J genetic background. Eight‐ to 16‐wk‐old mice were used in all experiments. All experiments were approved by the Local Ethics Committee (Uppsala djurförsöksetiska nämnd).

### 2.2 Cells, tumor inoculation, and tissue collection

The cell line B16.F10 (ATCC; CRL‐6475) was a gift from A.R. Thomsen (Copenhagen University, Denmark). Tumor cells were cultured in DMEM supplemented with 10% FBS, 1% L‐glutamine, and 1% penicillin and streptomycin solution. Prior to subcutaneous injections, cells reaching approximately 90%–100% confluency were trypsinized, resuspended in Hanks' balanced salt solution, and counted using trypan blue in order to adjust the cell concentration to 500,000 cells/ml. A total of 50,000 B16F10 cells (100 µl of cell suspension) were injected subcutaneously in the hip region (both sides). From day 7 post‐injection and every two days, the mice were examined for tumor growth. The size of tumors (a‐length, b‐width, tumor volume = (axb^2^)/2) was determined with a caliper, and the mice were sacrificed when the tumor volume reached 1,100 mm^3^ (all mice within an experimental group were sacrificed when one of the animals reached a tumor volume of 1,100 mm^3^). Blood was collected from B16F10 cell‐injected and naïve mice into 1.5‐ml microtubes and left to coagulate at room temperature for at least 1h. Blood samples were centrifuged at 2000g (4°C, 20 min), and serum was aliquoted and stored at −80°C for ELISA analysis. Tumors and inguinal lymph nodes were frozen on dry ice and stored at −80°C for gene array and qPCR analysis. Tumors were alternatively placed in 4% formalin (PBS‐buffered) solution for histological analysis and immunohistochemistry.

### 2.3 Histochemistry and immunohistochemistry

Sections from paraffin‐embedded tissue were deparaffinized and rehydrated, and epitope retrieval was performed by covering sections with 20 μg/ml of proteinase K in 50 mM Tris, pH 8.0, 1 mM EDTA, and 0.5% Triton X‐100 and incubated for 15 min at 37°C in a humidified chamber. Samples were brought to room temperature for 10 min, followed by rinsing with PBS‐T (10 mM phosphate buffer, pH 7.4, 2.7 mM KCl, 140 mM NaCl, and 0.1% Tween‐20) and blocking for 10 min with Background Sniper solution (Biocare Medical, Pacheco, CA) at room temperature. Incubation with rabbit anti‐CPA3 immune serum (1:500) (Rönnberg & Pejler, 2012) was performed overnight at 4°C, followed by addition of goat anti‐rabbit Alexa 633 (1:1,000) at room temperature for 1h. Rabbit anti‐Mcpt6 immune serum (1:500) (Rönnberg & Pejler, 2012) was added overnight at 4°C, followed by incubation with goat anti‐rabbit Alexa 488 (1:1,000) at room temperature for 1h. Controls were prepared in parallel by using immune serum at the same concentration as the primary immune serum. Samples were extensively rinsed between all steps with PBS‐T. ActinRed^TM^ 555 and NucBlue probes were used according to the manufacturer's instructions (Life Technologies, Carlsbad, CA). Slides were prepared using SlowFade^TM^ diamond antifade mounting medium (Invitrogen, Carlsbad, CA) and analyzed using a laser‐scanning microscope equipped with ZEN 2009 software (LSM 710; Carl Zeiss, Berlin, Germany). Staining of cultured B16F10 cells was performed as described (Rabelo Melo, Santosh Martin, Sommerhoff, & Pejler, 2019) Briefly, aliquots of 300 μl/well from 0.1∙10^6^ B16F10 cells/ml suspensions were cultured in 8 chamber polystyrene vessel tissue culture treated glass slides (Falcon, New York, NY) ± 50 nM recombinant Mcpt6. After 24h, supernatants were removed and cells were fixed with 4% paraformaldehyde in PBS for 15 min. One hundred microliters of 50 μg/ml digitonin solution in PBS was added to each individual glass and incubated for 10 min at room temperature, followed by washing three times with TBS‐T. The slides were treated with 300 μl of rabbit anti‐Mcpt6 immune sera (1:500) (Rönnberg & Pejler, 2012) overnight (4°C), followed by incubation with goat anti‐rabbit Alexa 488 (1:1,000) (Invitrogen, Rockford, IL) at room temperature for 1h. The slides were rinsed 3 × with TBS‐T, and then, ActinRed^TM^ 555 and NucBlue probes were added according to the manufacturer's instructions (Life Technologies, Carlsbad, CA). Slides were prepared using SlowFade^TM^ diamond antifade mounting medium (Invitrogen, Carlsbad, CA) and analyzed using a laser‐scanning microscope equipped with ZEN 2009 software (LSM 710; Carl Zeiss, Berlin, Germany).

### 2.4 Recombinant Mcpt6 and EdU staining

Mcpt6 was produced in the mammalian cell line HEK293‐EBNA with the vector pCEP‐Pu2 according to previously published procedures (Hallgren, Karlson, Poorafshar, Hellman, & Pejler, 2000; Vernersson, Ledin, Johansson, & Hellman, 2002). The coding regions for full‐length enzymes containing an N‐terminal 6‐histidine purification tag and the proteins were purified from conditioned media from transfected HEK293‐EBNA cells on Ni‐chelating IMAC agarose (Qiagen, Hilden Germany). Following activation cleavage, the enzyme was activated by adding heparin and lowering the pH as previously described (Fu, Akula, Thorpe, & Hellman, 2019; Hallgren et al., 2000). EdU staining and flow cytometry were performed as described (Rabelo Melo et al., 2019).

### 2.5 RNA extraction, quantitative RT‐PCR (qPCR), and gene array analysis

Mice were euthanized, and tumors were collected, frozen on dry ice, and stored at −80°C until use. Tissues were homogenized in TRIzol reagent (Thermo Scientific, Wilmington, DE) using a polytron PT 1200 (Kinematica AG, Luzern, Switzerland). The homogenate was centrifuged at 12,000 *g* for 1 min, and 500 µl of the supernatant (corresponding to ~50 mg tissue) was used for total RNA isolation using the Direct‐zol RNA MiniPrep Kit (The Epigenetics Company, Irvine, CA). Total RNA concentration and purity were measured using a NanoDrop 1000 Spectrophotometer (Thermo Scientific, Wilmington, DE) and the ND‐1000 V3.7.0 program. First‐strand cDNA was synthesized using up to 1 µg of total RNA as template and the iScript cDNA synthesis kit (Bio‐Rad, Hercules, CA), following the manufacturer's instructions, on a SimpliAmp Thermal Cycler instrument (Applied Biosystems by Life Technologies/Thermo Fisher Scientific, Darmstadt, Germany). Subsequently, qPCR was performed using up to 100 ng cDNA, 400 nM primers (indicated in Supporting Table S1) and iTaq Universal SYBR Green Supermix (Bio‐Rad, Hercules, CA), following the PCR cycling conditions recommended by the manufacturer, on the C1000 Touch Thermal Cycler instrument (Bio‐Rad, Hercules, CA). Each sample was run in duplicates/triplicates, and qPCR data analysis was performed using the Bio‐Rad CFX Maestro program. Gene expression levels were presented relative to the house keeping gene (glyceraldehyde 3‐phosphate dehydrogenase; GAPDH) and relative either to WT inoculated mice or to respective non‐inoculated naïve mice.

For analysis of miR3098 and miR669b, first‐strand cDNA was synthesized using Qiagen miRCURY LNA RT kit (cat.# 339340) followed by qPCR with the Qiagen miRCURY LNA SYBER Green PCR kit (cat.# 339345) and miRCURY LNA miRNA PCR assays (primers) specified in Supporting Table S1. qPCR samples were run in duplicates, and gene expression levels were presented relative to non‐coding 5S‐rRNA and relative to WT inoculated mice.

Gene array analysis was performed using Affymetrix GeneChip1 expression arrays (GeneChip1 Mouse Gene 1.0 ST Array), as described previously (Rönnberg, Guss, & Pejler, 2010).

### 2.6 ELISA

Mouse CXCL9 ELISA (cat.# ab203364, Abcam, Cambridge, UK) and mouse IFNγ ELISA (cat.# BMS609, Thermo Scientific, Wilmington, DE) were performed with serum from naïve mice or from B16F10 cell‐injected mice. Absorbance was determined with a microplate reader: Tecan Infinite 200 (Tecan Austria, Grödig, Austria) and the Magellan V. 6.6 software.

### 2.7 Statistical analysis

All analyses were performed in GraphPad Prism using two‐tailed unpaired test, Mann–Whitney, 2‐way ANOVA with Tukey's multiple comparison test (cell populations), multiple *t* test (cell populations), and unpaired *t* test (EdU labeling experiments, cell numbers). Results shown are either from representative experiments or represent collected data from at least 2 independent experiments, presented as mean values ± *SEM*. A *p* value ≤ .05 was considered statistically significant.

## 3 RESULTS

### 3.1 Tumors develop more rapidly in Mcpt6^−/−^ than in WT mice

**Figure 1:**
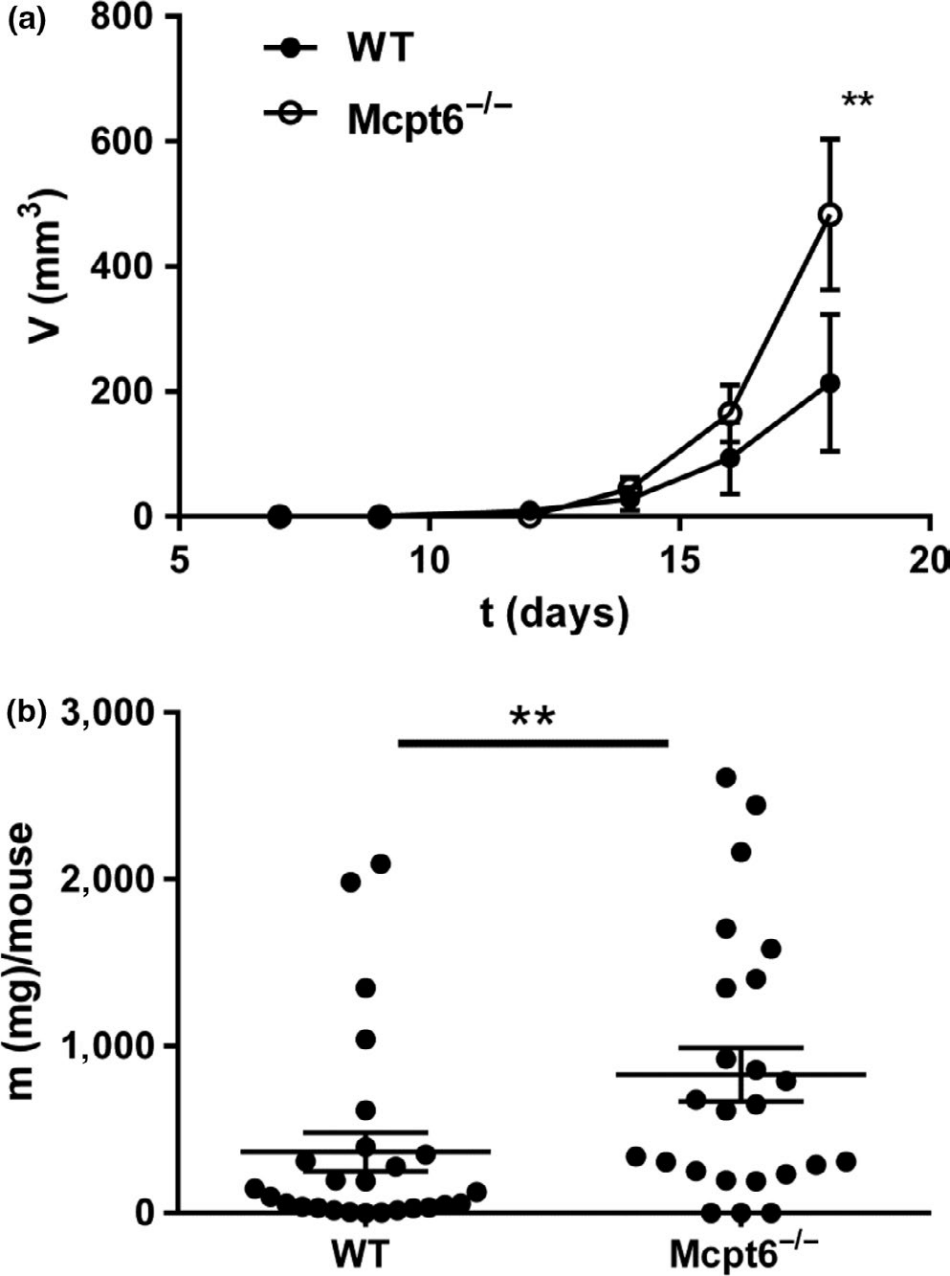
Mcpt6‐deficient mice develop larger tumors than WT mice. (a) 50,000 B16F10 cells were injected subcutaneously in the hip region of WT and Mcpt6‐deficient mice. From day 7 post‐injection and every two days, the mice were examined for tumor growth. Tumor volumes are presented as mean values ± *SEM* (*n* = 5–19). Note the larger volumes of tumors in Mcpt6‐deficient versus WT mice on d.18 post‐B16F10 injection. (b) Weight of tumors dissected after termination of the experiments. Note the larger mass of tumors from Mcpt6^−/−^ versus WT mice. Tumor mass is presented as mean values ± *SEM* (*n* = 24–26). **p ≤ .01

To study the impact of tryptase on tumor progression, we injected melanoma cells (B16F10) into the flanks of WT and tryptase null (Mcpt6^−/−^) mice. Tumor progression was then followed. As seen in Figure 1a, palpable tumors appeared starting from day ~7 in both WT and Mcpt6^−/−^ mice. However, the tumors developed markedly more rapidly in Mcpt6^−/−^ mice in comparison with WT controls, as quantified by continuous measurements of tumor volume in live animals. An increased tumor size in Mcpt6^−/−^ versus WT animals was confirmed after dissecting out and weighing the tumors (Figure 1b).

### 3.2 MCs are present in the stroma of subcutaneous melanoma tumors

**Figure 2:**
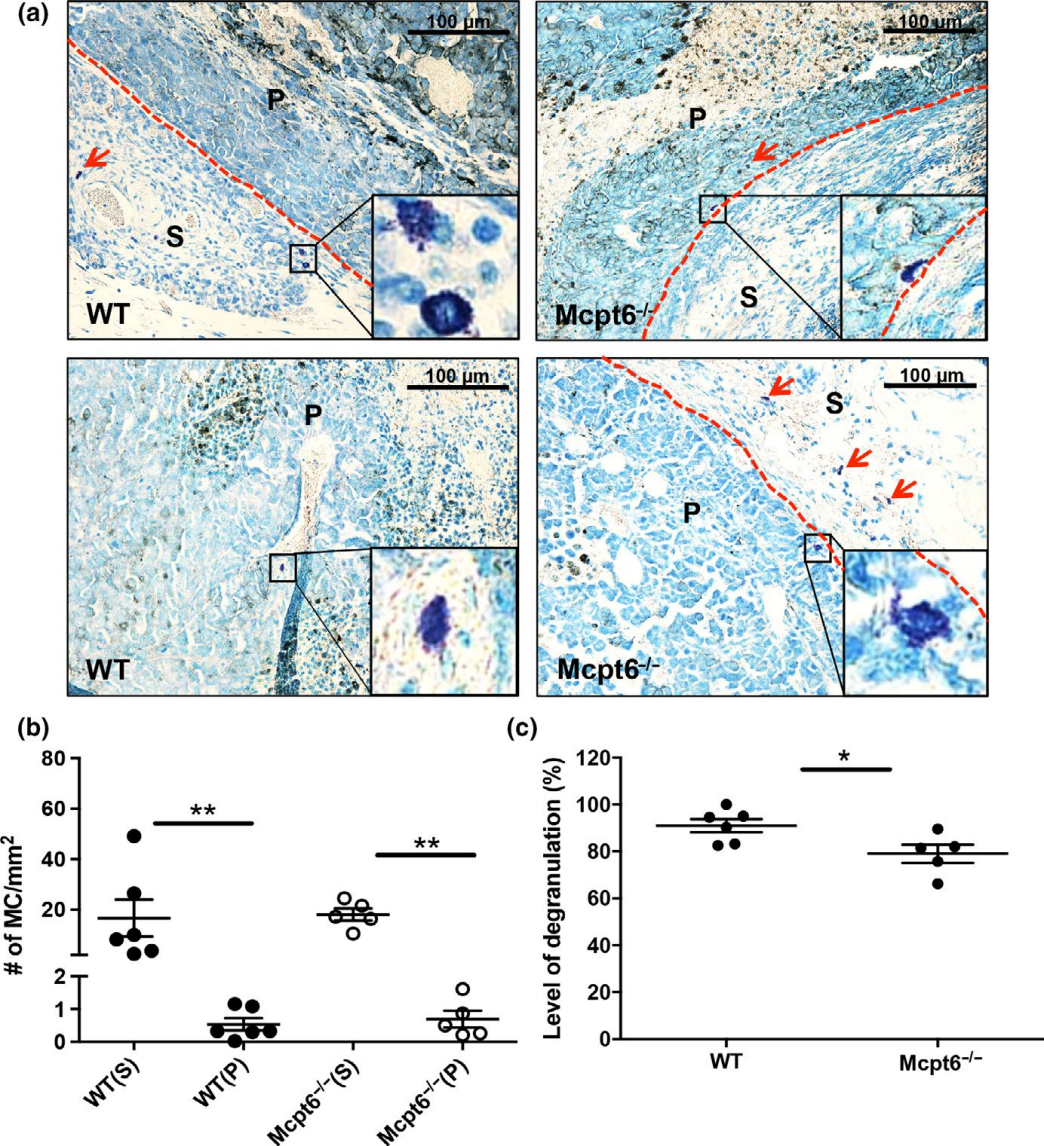
MCs are predominantly present in the tumor stroma. Tumors were paraffin‐embedded, sectioned, and stained with toluidine blue. (a) Representative images showing the presence of toluidine blue‐positive MCs (marked by arrows) both in the tumor stroma (S) and in the tumor parenchyma (P) (red dashed line separating P and S areas). Inserts show a higher magnification of toluidine blue‐stained MCs. Original magnification × 200; bars = 100 µm. (b) Quantification of toluidine blue‐positive MCs. Numbers of MCs/mm^2^ are presented as mean values ± *SEM* (*n* (tumor) = 5); **p ≤ .01. Note that the majority of the MCs were detected in the tumor stroma and that tumors from WT and Mcpt6^−/−^ mice had comparable numbers of MCs. (c) Numbers of MCs showing signs of degranulation in tumors from WT and Mcpt6^−/−^ mice. *p ≤ .05

To approach how MCs impact on the subcutaneous melanoma tumors, we first assessed the presence and location of MCs in the tumors. To identify MCs, we used toluidine blue staining. As seen in Figure 2a,b, MCs were indeed found in the tumors. Notably, they showed a preferential location to the tumor stroma but could also be found within the tumor parenchyma. As judged by the toluidine blue staining, a substantial fraction of the MCs showed signs of degranulation, indicating that MCs had been activated within the tumor setting (Figure 2c). Importantly, MCs were equally abundant in tumors formed in WT and Mcpt6^−/−^ mice, indicating that the impact of Mcpt6 on tumor growth is not associated with differential migration of MCs into the tumor (Figure 2). Moreover, as in WT mice, the MCs in tumors of Mcpt6^−/−^ mice showed a preferential location to the tumor stroma as judged by toluidine blue staining (Figure 2).

### 3.3 Melanoma‐associated MCs express Mcpt6

**Figure 3:**
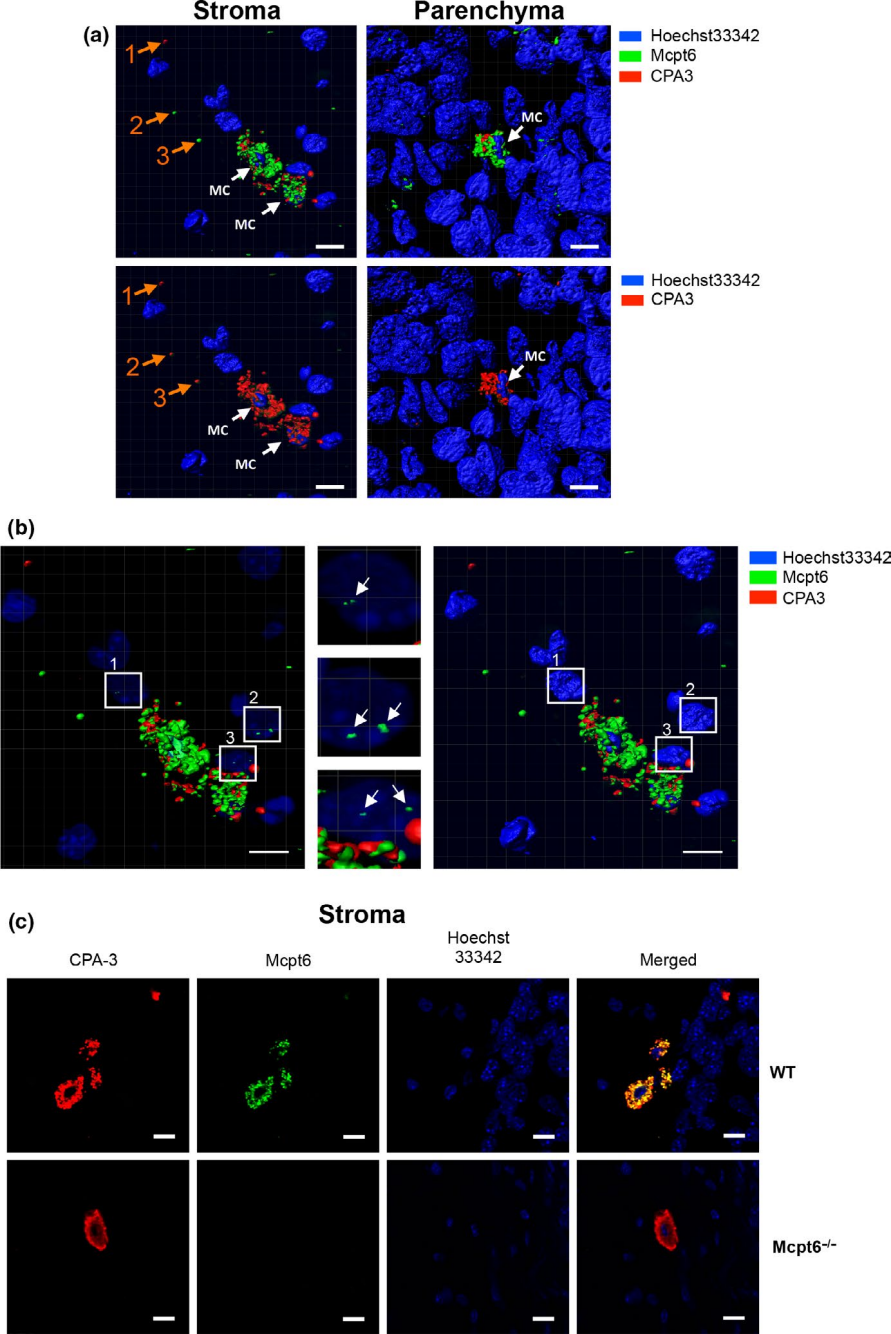
Melanoma‐associated MCs are of connective tissue subtype and release tryptase‐containing granules to the tumor microenvironment. Confocal microscopy analysis of tumors from WT and Mcpt6^−/−^ mice. (a) 3D images generated from z‐stack tumor sections of WT mice showing the presence of MCs in the tumor stroma and parenchyma. The upper panels show Mcpt6 and CPA3 staining together, and the lower panels show CPA3 staining only. MCs are indicated by white arrows. Examples of individual granules are indicated by orange arrows. Note that the majority of MCs granules are Mcpt6 and CPA3 double‐positive and are widely distributed within the tumor tissue. (b) Mcpt6 can be found within the nucleus of tumor cells. The left panel depicts translucent nuclear staining showing the presence of Mcpt6 within the nucleus of tumor cells adjacent to MCs. The middle panel represents higher magnifications of the boxed regions in the left panel and highlights the nuclear localization of Mcpt6 within tumor cells (see also Supporting Video S1). In the right panel, the nuclear staining is shifted into solid blocks; note that the Mcpt6 signal thereby is lost (Supporting Video S1). (c) WT melanoma‐associated MCs are double‐positive for Mcpt6 and CPA3, indicating that they are of connective tissue subtype. The lower panels depict staining for Mcpt6 in tumors from Mcpt6^−/−^ mice; note that the Mcpt6 staining is completely abolished, revealing that the staining is specific. Scale bars = 10 μm

In mice, MCs are subdivided into two major subclasses—mucosal type MCs (MMCs) and connective tissue‐type MCs (CTMCs). MMCs express chymases only (Mcpt1, Mcpt2), whereas CTMCs express tryptases (Mcpt6, Mcpt7), chymases (Mcpt4, Mcpt5), and CPA3 (Pejler, Åbrink, Ringvall, & Wernersson, 2007; Pejler et al., 2010). Since our data suggest that Mcpt6 has an impact on melanoma growth, we hypothesized that the tumor‐associated MCs were of CTMC type, that is, expressing Mcpt6. Indeed, as shown by confocal microscopy analysis, the tumor‐associated MCs in WT mice were strongly positive for Mcpt6 (Figure 3). They were also positive for CPA3, confirming that they were of CTMC subtype. As expected, MCs in tumors of Mcpt6^−/−^ mice did not express Mcpt6 protein. However, they showed CPA3 positivity, hence indicating that the tumor‐associated MCs in Mcpt6^−/−^ mice were also of CTMC subtype (Figure 3c). It was also notable that Mcpt6 and CPA3 were released in granule‐like structures from the MCs into the surrounding milieu. Notably, most of the released granules were double‐positive for Mcpt6 and CPA3, suggesting that these proteases are present in the same types of granules (Figure 3a). Altogether, these findings indicate that the tumor‐infiltrating MCs in both WT and Mcpt6^−/−^ mice were of the CTMC subtype and that they release granule‐contained proteases into the microenvironment.

### 3.4 Mcpt6 is released from tumor‐infiltrating MCs and enters melanoma cells

**Figure 4:**
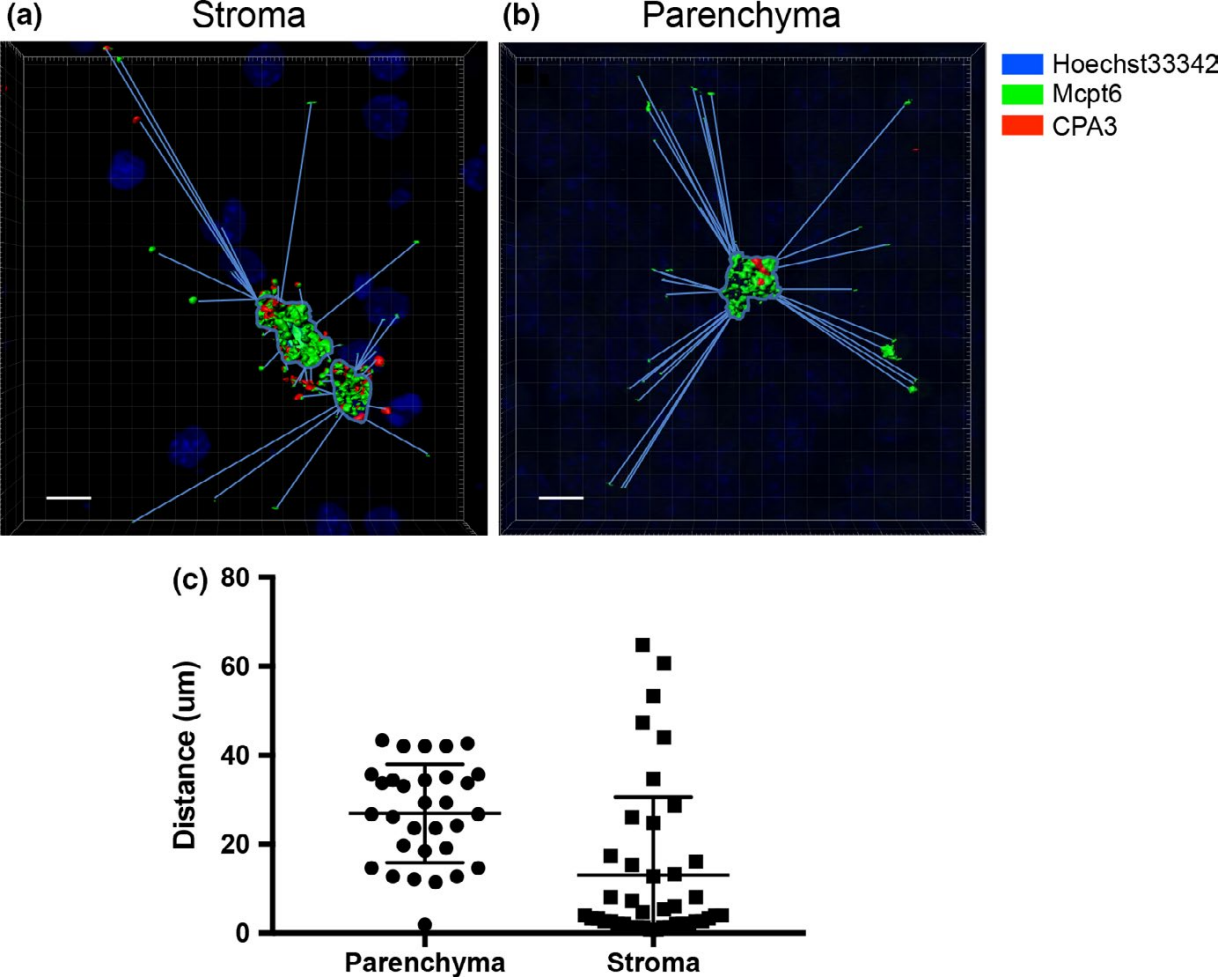
Mcpt6^+^ granules can be found at a large distance from MCs in the tumor stroma and parenchyma. 3D images from Z‐stack confocal sections of tumor stroma (a) and parenchyma (b) were used to measure the distance between Mcpt6^+^ granules and the closest located MC. (c) Distances between MCs and individual granules were measured in µm. Scale bars = 10 μm

As judged by toluidine blue staining, the tumor‐associated MCs were predominantly located in the tumor stroma and were poorly visible in the tumor parenchyma (Figure 2). However, when using confocal microscopy, MCs were more readily detected within the tumor parenchyma (Figure 3a). Similar to the MCs in the stroma, these MCs were double‐positive for Mcpt6 and CPA3, indicating that they were of CTMC subtype. An intriguing observation was that Mcpt6 was released from the parenchymal MCs, and was widely spread within the tumor parenchyma. An assessment of the distance between released Mcpt6^+^ granules and tumor‐associated MCs revealed that granules can be found up to ~40 µm from the closest located MCs within the tumor parenchyma and up to ~60 µm from the closest located MCs in the tumor stroma (Figure 4). Moreover, the confocal analysis suggested that the released Mcpt6 was in fact found inside the tumor cells, partially localized to the tumor cell nuclei (Figure 3b; Supporting Video S1). CPA3 was also released from the MCs populating the tumor parenchyma, although the extent of release and uptake into tumor cells was less pronounced (Figure 3b).

### 3.5 Recombinant Mcpt6 is taken up by melanoma cells and causes reduced proliferation

**Figure 5:**
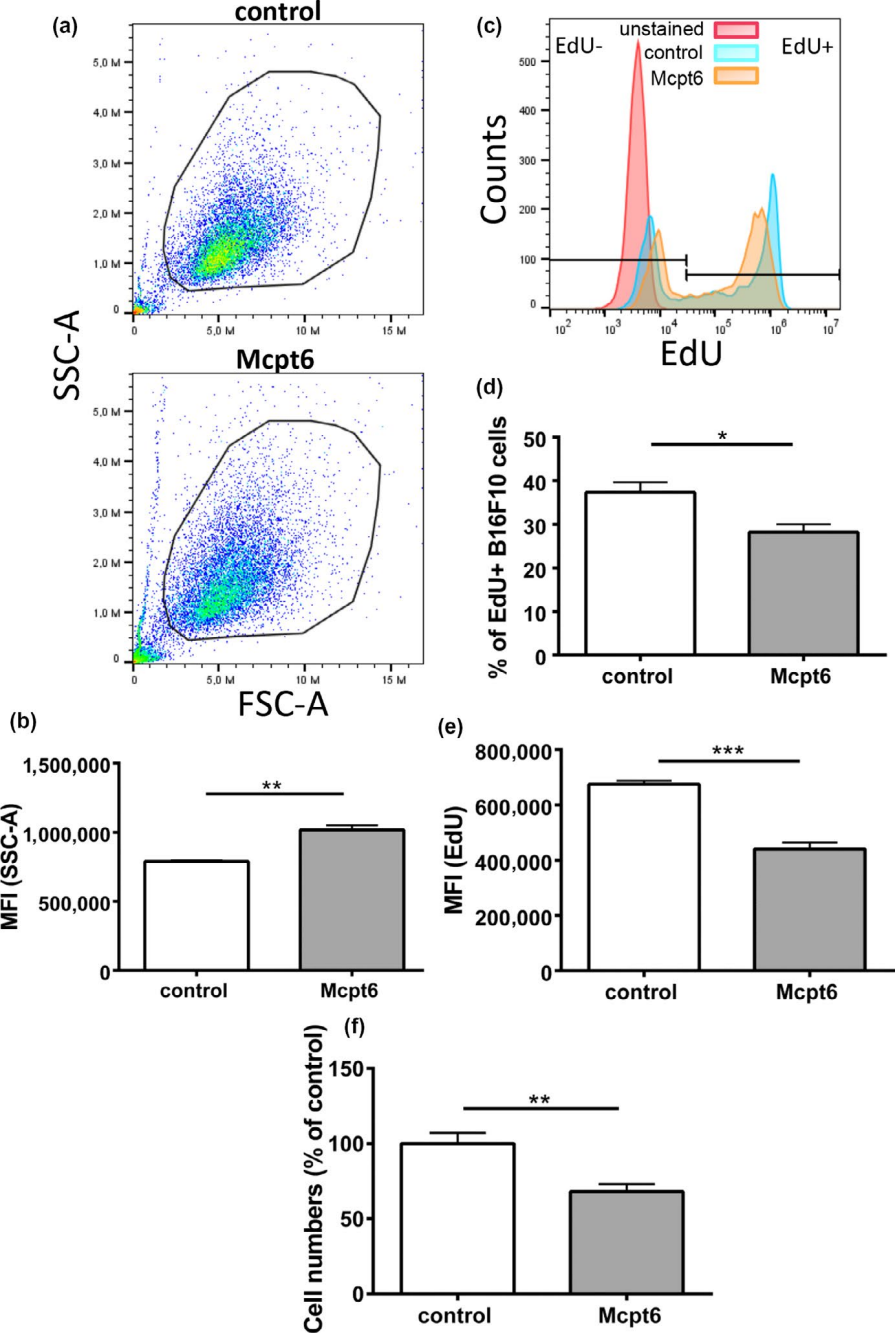
Mcpt6 inhibits proliferation of B16F10 cells. B16F10 melanoma cells were cultured for 48h in the presence of 50 nM recombinant Mcpt6. (a‐b) Representative dot blots show gated alive cells and reveal that B16F10 cells exposed to Mcpt6 show enhanced granularity, presented by higher mean fluorescence intensity (MFI; SSC‐1). (b) Quantification of SSC analysis. The data represent mean values ± *SEM* from one representative experiment (*n* = 3); unpaired *t* test; **p ≤ .01. (c‐e) Cells were assessed for proliferation by EdU staining and flow cytometry analysis. (c) Merged representative histograms for the EdU staining. (d‐e) Quantification of the EdU staining, given as % EdU^+^ cells (d) and MFI for the EdU staining (e). The data represent mean values ± *SEM* (*n* = 6 [d]; *n* = 3 [e]); unpaired *t* test; *p ≤ .05, ***p ≤ .001. (f) Quantification of cell numbers using a hemocytometer. Data are presented as mean values ± *SEM*, pooled from 2 independent experiments; analyzed with unpaired *t* test; **p ≤ .01

**Figure 6:**
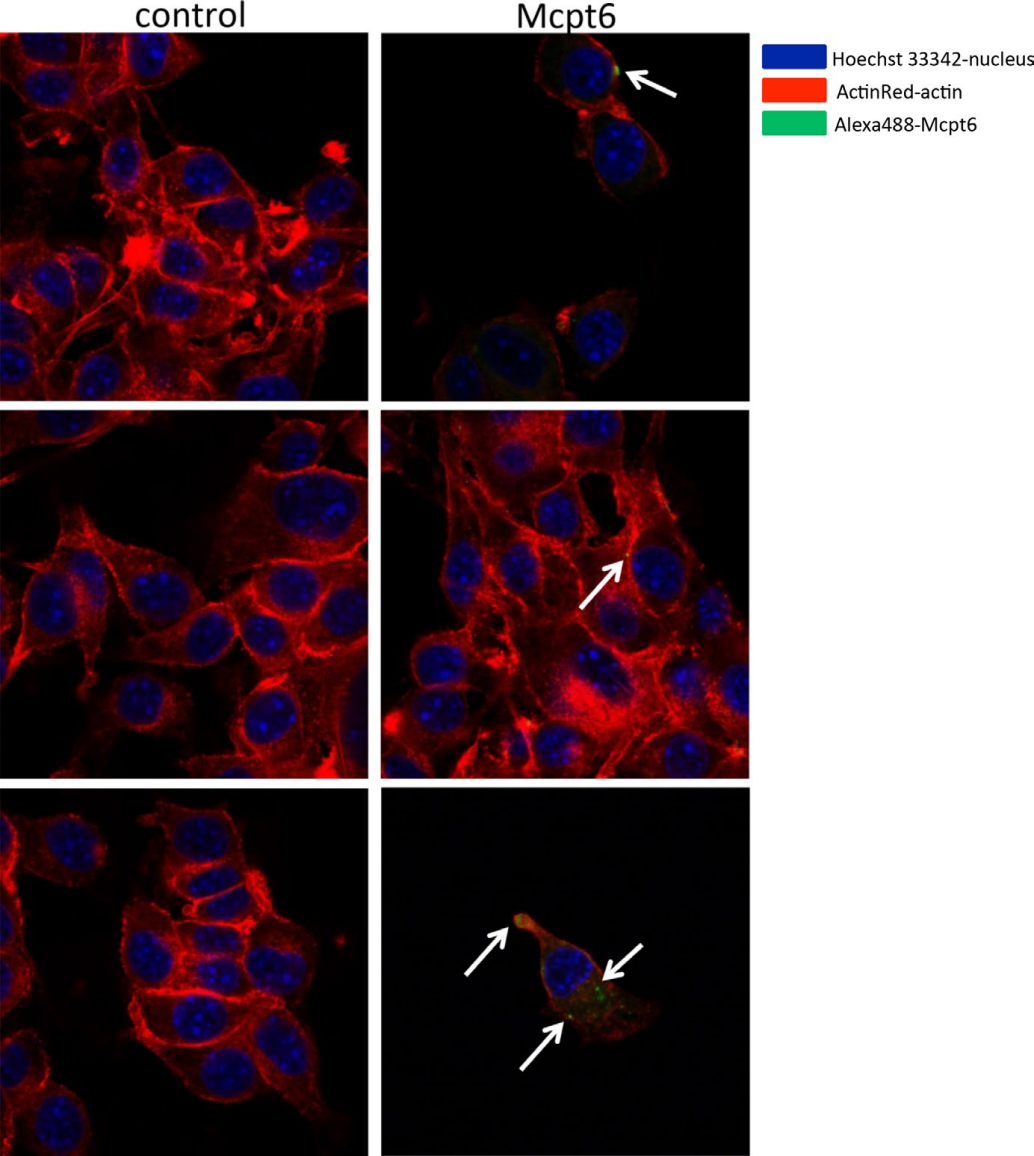
Recombinant Mcpt6 is taken up by melanoma cells. B16F10 melanoma cells were cultured with or without 50 nM recombinant Mcpt6 as indicated. After 24h, the cells were stained with anti‐Mcpt6 antibody and were analyzed by confocal microscopy. Cells were co‐stained using a nuclear marker (Hoechst 33342; blue) and an actin probe (ActinRed; red). The figure depicts representative images. White arrows indicate positive Mcpt6 staining. Note that Mcpt6 staining is seen at the cell surface and also in the cytoplasm (lower panel), the latter indicating uptake of Mcpt6. Note also that no positive staining is seen in the absence of added Mcpt6 (control; left panels)

To provide further mechanistic insight into how tryptase affects melanoma cells, we generated recombinant Mcpt6 and assessed how it affects cultured B16.F10 melanoma cells. As seen in Figure 5a,b, addition of recombinant Mcpt6 to the melanoma cells caused morphological effects, as judged by an increase in the side scatter parameter upon flow cytometry analysis. To assess proliferation, we used EdU, a thymidine analogue. By using this approach, we found that recombinant Mcpt6 causes decreased EdU staining of the melanoma cells, both as regards the % of EdU^+^ cells and with regard to the mean fluorescence intensity of the EdU staining (MFI) (Figure 5c–e). Hence, this indicates that Mcpt6 causes reduced proliferation of the melanoma cells. Reduced melanoma cell proliferation after exposure to recombinant Mcpt6 was also supported by assessment of cell numbers (Figure 5f). To assess the potential physical interaction between recombinant Mcpt6 and the melanoma cells, we performed confocal microscopy analysis. This showed that the melanoma cells, after exposure to recombinant Mcpt6, frequently were positive for Mcpt6 and that Mcpt6 could be found both close to the cell membrane and in the cell interior (Figure 6). Together, these data indicate that recombinant Mcpt6 interacts with and is taken up by melanoma cells, and causes reduced melanoma cell proliferation.

### 3.6 Mcpt6 affects gene expression in the tumors

**Figure 7:**
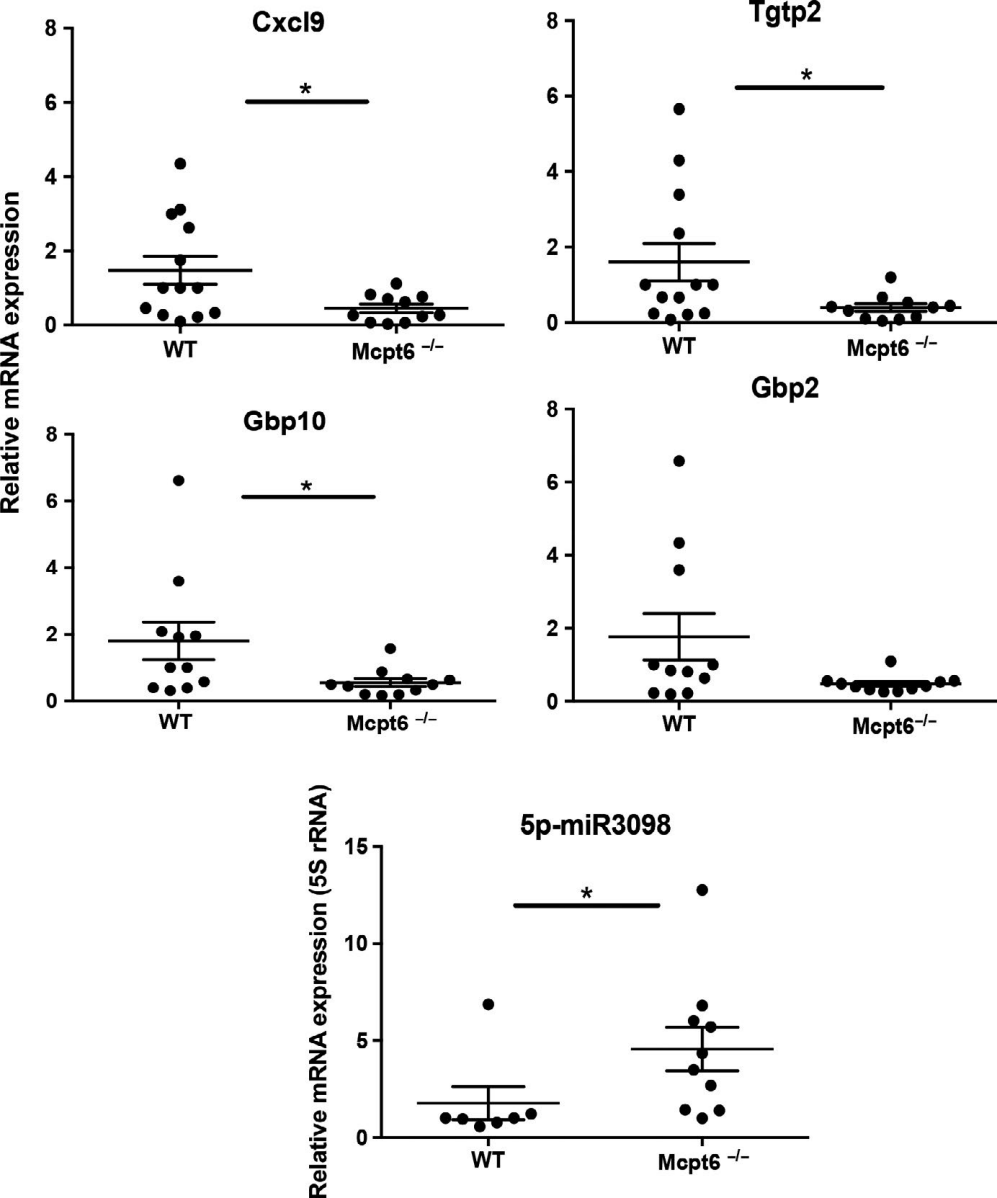
Mcpt6 deficiency affects gene expression in melanoma tumors. Total RNA was isolated from tumors of WT and Mcpt6‐deficient mice. Subsequently, total RNA was subjected to qPCR analysis of expression of *Cxcl9*, *Tgtp2*, *Gbp10*, *Gbp2*, and miR3098. Expression of genes was evaluated relative to either GAPDH (protein‐encoding genes) or 5S‐rRNA (miRNAs), and normalized to WT mice. Note the increased expression of *Cxcl9, Tgtp2,* and *Gbp10*, and lower expression of miR3098 in tumors from WT versus Mcpt6^−/−^ mice. Results are presented as mean values ± *SEM* (*n* = 7–13); Mann–Whitney test. *p ≤ .05

**Figure 8:**
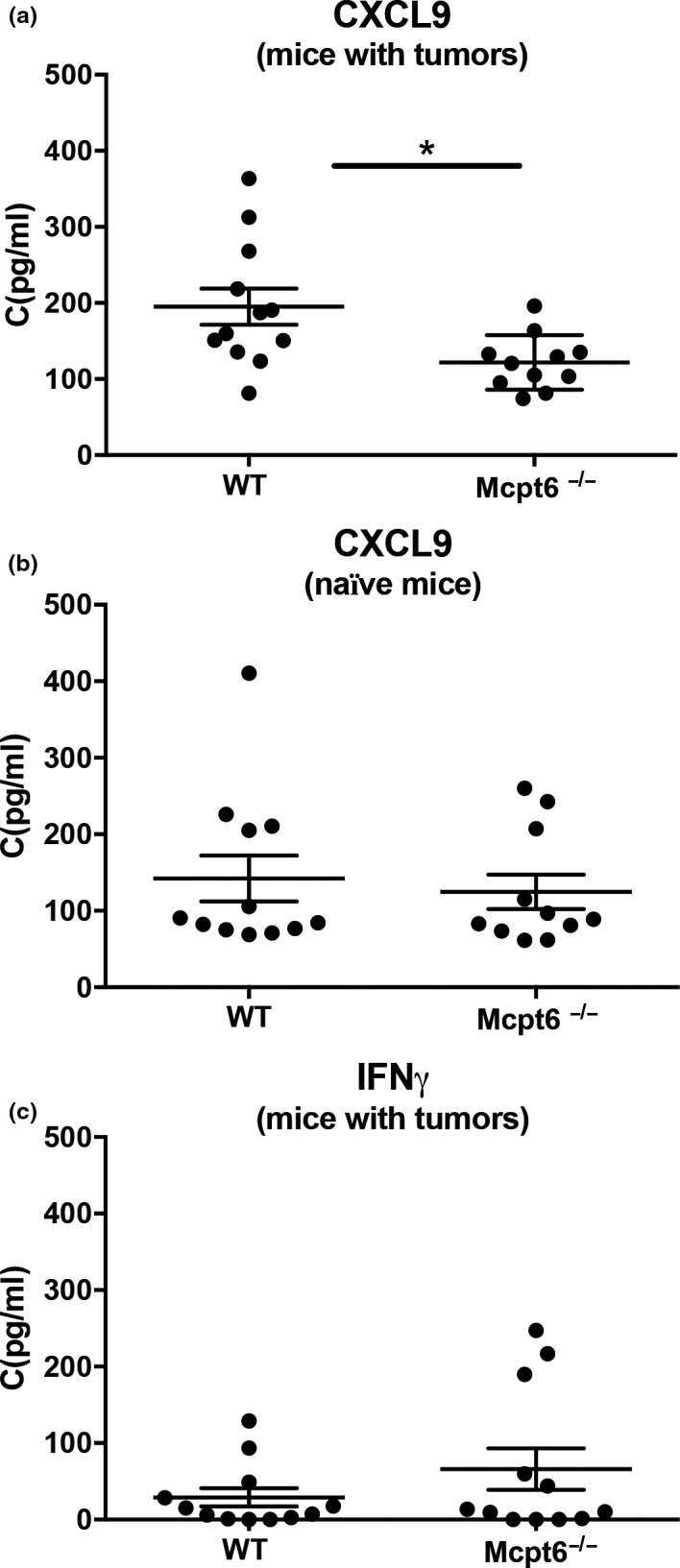
Higher levels of CXCL9 but not of IFNγ in serum of WT compared to Mcpt6^−/−^ mice. 50,000 B16F10 cells were injected subcutaneously in the hip region of WT or Mcpt6^−/−^ mice. After termination of the experiment, blood was collected. Blood was also collected from naïve WT and Mcpt6^−/−^ mice. (a‐b) Levels of CXCL9 in serum from (a) B16F10‐injected (*n* = 12) and (b) naïve (*n* = 11) mice, measured by ELISA. Note the higher levels of CXCL9 in serum from B16F10‐injected WT versus Mcpt6^−/−^ mice, whereas no differences in CXCL9 levels were seen between WT and Mcpt6^−/−^ naïve mice. (c) IFNγ levels in serum from B16F10‐injected WT and Mcpt6^−/−^ mice, measured by ELISA (*n* = 12). Results are presented as mean values ± *SEM*; Mann–Whitney test. *p ≤ .05

To further address the mechanism behind the impact of Mcpt6 on tumor growth, we hypothesized that Mcpt6 might affect gene expression patterns in the tumors. This was prompted by our previous observations that Mcpt6 can have marked effects on gene expression in MCs (Melo et al., 2017). To evaluate this possibility, we prepared RNA from multiple tumors of WT and Mcpt6^−/−^ mice, pooled these, and subjected the samples to gene array analysis. Indeed, this screening suggested that a number of genes might be differently regulated in tumors from WT versus Mcpt6^−/−^ mice (Supporting Tables S2 and S3). To verify and quantify these data, we performed qPCR analysis, by focusing on a number of genes of interest. These included the IFNγ‐dependent genes *Cxcl9, Gbp2,* and *Gbp10*, as well as *Tgtp2*. qPCR analysis showed that all of these genes were significantly higher expressed in WT versus Mcpt6^−/−^ tumors. There was also a trend of higher *Gbp2* expression in WT versus Mcpt6^−/−^ tumors. In contrast, the IFNγ gene was not differently expressed (Figure 7). The gene array screen also indicated that a number of microRNAs were differently regulated as a consequence of Mcpt6 deficiency. Indeed, qPCR analysis confirmed that one of these, 5p‐miR3098, was significantly lower expressed in tumors from WT versus Mcpt6^−/−^ mice (Figure 7). To verify the effect on *Cxcl9* at the protein level, ELISA analysis was conducted. In agreement with the gene array and qPCR data, we found that CXCL9 levels were significantly higher in serum from WT versus Mcpt6^−/−^ mice (Figure 8). We also measured IFNγ levels in serum, but found no significant difference between WT and Mcpt6^−/−^ animals (Figure 8).

**Figure 9:**
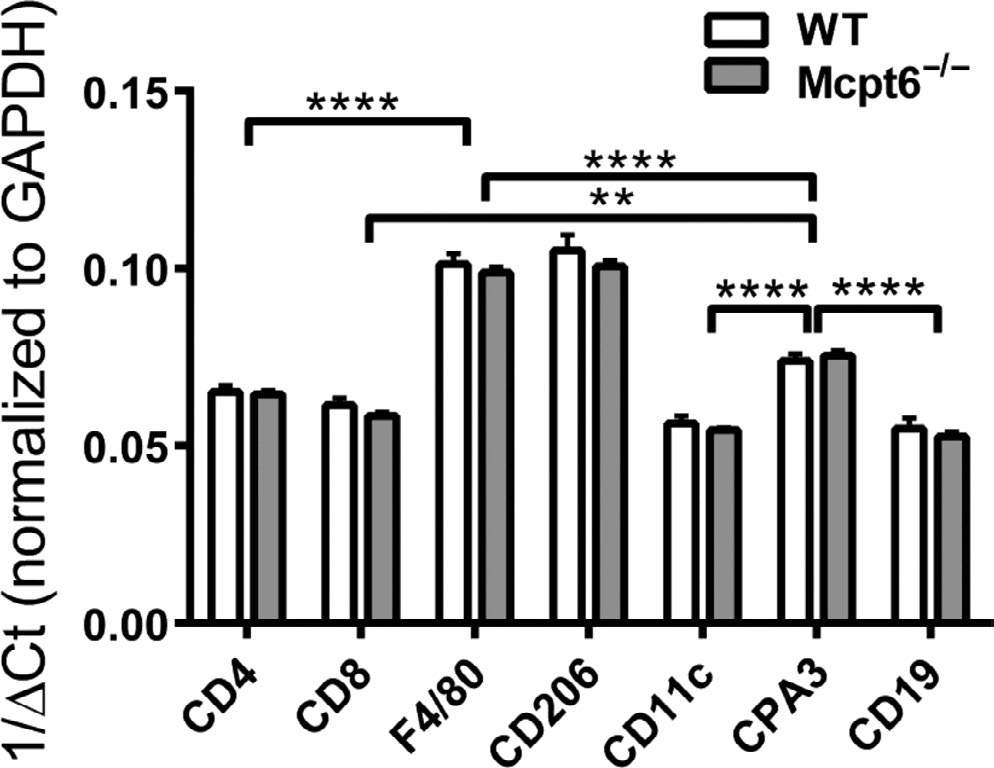
qPCR analysis of tumors for the expression of the cell‐specific markers CD4, CD8, F4/80, CD11c, CD206, CD19, and CPA3. Total RNA was isolated from tumors of WT and Mcpt6^−/−^ mice. Total RNA was subjected to qPCR analysis for expression of the CD4, CD8, F4/80, CD11c, CD206, CD19, and CPA3 genes. Gene expression was evaluated relative to GAPDH (housekeeping gene) and presented as 1/∆Ct. Note that the qPCR analysis indicated that all of these populations were equally represented in WT and Mcpt6^−/−^ mice. Results are presented as mean values ± *SEM* (*n* = 9–11). **p ≤ .01; ****p ≤ .0001

Next, we used qPCR analysis to evaluate whether the absence of Mcpt6 affected different populations of tumor‐infiltrating immune cells. This was assessed by analyzing for cell‐specific markers: CD4 (CD4 T cells), CD8 (CD8 T cells), F4/80 (macrophages), CD206 (type 2‐polarized macrophages), CD11c (type 1‐polarized macrophages), CD19 (B cells), and CPA3 (MCs). However, none of these genes were differently regulated in tumors from WT versus Mcpt6^−/−^ mice, indicating that Mcpt6 does not affect the profile of immune cells infiltrating the melanomas (Figure 9). As judged by the ΔCt values for the respective genes, type 2‐polarized macrophages (CD206^+^) appeared to be abundant within the tumors.

## 4 DISCUSSION

The bona fide role of MCs in malignancies is still enigmatic. Intriguingly, their role may vary considerably among different types of cancers and perhaps also in the various stages of a malignant condition (Varricchi et al., 2017). Clearly, since a profound MC accumulation is evident in essentially all investigated malignant settings (Marichal et al., 2013; Oldford & Marshall, 2015; Ribatti & Crivellato, 2009; Varricchi et al., 2017), it is reasonable to assume that they indeed play a significant role. It is notable that MCs are often among the first types of immune cells that arrive at a malignant lesion (Dalton & Noelle, 2012; Oldford & Marshall, 2015), and we may thus envision that effects of MCs are of particular importance in the early phases of cancer development. However, MCs are consistently found also in fully developed cancers and are thereby likely to affect also late stages of malignant processes. As regards the mechanistic impact of MCs on cancer, a favored notion is that MCs can contribute by expressing cancer‐driving pro‐angiogenic factors such as VEGF, FGF2, angiopoietin‐1, and matrix metalloproteinases (Baram et al., 2001; Esposito et al., 2004; Imada, Shijubo, Kojima, & Abe, 2000; Nakayama, Yao, & Tosato, 2004; Ribatti, Vacca, et al., 2003; Toth‐Jakatics et al., 2000). Indeed, a correlation between the MC expression of such factors and cancer progression has been noted (Ribatti, Vacca, et al., 2003; Toth‐Jakatics et al., 2000). However, it remains to be demonstrated by using direct experimental approaches (e.g., MC‐conditional deletion of the respective genes) that MCs affect malignant development by affecting angiogenesis.

In this study, we show that MC tryptase, Mcpt6, affects the outcome in a subcutaneous model of melanoma. Our data indicate that Mcpt6, under the experimental conditions employed, has a protective role, which is in contrast with the more common view of MCs as detrimental players in melanoma and other cancers. However, it is notable that our findings are in line with a previous study where we showed that the collective absence of all of the proteases expressed by connective tissue‐type MCs led to a higher colonization of lungs with melanoma cells following their i.v. administration (Grujic et al., 2017). Moreover, our findings are consistent with clinical evidence in support of a correlation between MC protease expression and protection against melanoma (Crincoli et al., 2019; Siiskonen et al., 2015; Stieglitz et al., 2019). A protective role of MCs is also supported by a study where secreted MC mediators showed anti‐proliferative effects on cultured melanoma cells, although the nature of the active MC mediator(s) was not revealed (Stieglitz et al., 2019).

An important issue is to define how MC tryptase exerts its protective function in melanoma. To address this, we first assessed whether MCs were present in the melanomas, as well their location within the tumor and their phenotype. In agreement with many previous studies, our findings reveal that MCs are predominantly detected in the tumor stroma, but were more rare in the tumor parenchyma by conventional staining techniques. However, when instead using confocal microscopy assessment with an anti‐Mcpt6 antibody, we found that MCs were in fact relatively abundant also within the tumor parenchyma. An important implication of these data was also that the tumor‐associated MCs, both in the stroma and within the parenchyma, express tryptase. Since they also expressed CPA3, this indicates that the tumor‐associated MCs are of CTMC rather than MMC subtype. This is thus in agreement with a previous study where it was shown that the tumor‐infiltrating MCs in glioblastoma were of CTMC type (Polajeva et al., 2011).

Another important observation, from both conventional and confocal microscopy assessment, was that the tumor‐associated MCs frequently showed signs of degranulation, hence suggesting that they release their preformed granule compounds into the tumor microenvironment. Further, an intriguing observation was that tryptase‐positive granules were widely distributed within the tumor, not only in the close vicinity to MCs. This suggests that MCs have the capacity to influence tumor cells that are located both in their close proximity but also at a larger distance. Clearly, this can explain why MCs, despite being relatively rare within tumors, can have an impact on the overall tumor progression.

Intriguingly, tryptase released from MCs in the tumor parenchyma was frequently found within the tumor cells, that is, indicating cellular uptake. This was also supported by our in vitro approach, where it was revealed that recombinant Mcpt6 is taken up by cultured melanoma cells. In an earlier study, we showed that Mcpt6 can have an impact on gene expression in MCs (Melo et al., 2014) and we also showed recently that human tryptase impacts on gene expression in human melanoma cells (Rabelo Melo et al., 2019). Prompted by these findings, we evaluated whether Mcpt6 can affect gene expression patterns within melanoma tumors. Indeed, we found that a number of genes were differently expressed in tumors lacking Mcpt6. Among these were genes associated with IFNγ signaling, including *Cxcl9* and the intracellular GTPase *Gbp10*. Increased *Cxcl9* expression was also supported by protein analysis, revealing higher levels of CXCL9 protein in serum from WT versus Mcpt6^−/−^ tumor‐bearing animals. CXCL9 is implicated as a tumor suppressor, due to its ability to recruit CD8^+^ cells (Harlin et al., 2009). However, we did not see any apparent impact of Mcpt6 deficiency on CD8 expression in the tumors, suggesting that any impact of CXCL9 on tumor progression may be explained by effects beyond its influence on CD8^+^ T‐cell recruitment. To date, the role of mouse Gbp10, one of eleven members of the Gbp family, in tumor biology is unclear. However, in human cutaneous melanoma, several Gbp mRNAs are associated with favorable prognosis (Wang et al., 2018). Hence, the higher expression of *Gbp10* in WT versus Mcpt6^−/−^ mice may represent a protective function in melanoma development. We also found upregulated expression of an additional GTPase, *Tgtp2*, in WT versus Mcpt6‐deficient animals. However, the role of *Tgtp2* in tumor settings remains to be explored. We also found that the absence of Mcpt6 was associated with elevated expression of the micro RNA miR3098. Interestingly, miR3098 has previously been implicated in lung cancer (Wang, Xu, & Wang, 2017) and diabetes (Rubin, Salzberg, Imamura, Grivitishvilli, & Tombran‐Tink, 2016), although the exact role miR3098 under these conditions has not been clarified.

To provide further mechanistic insight into how tryptase affects the melanoma cells, we performed experiments in which the effect of recombinant Mcpt6 on cultured melanoma cells was assessed. Interestingly, these analyses revealed that Mcpt6 caused a substantial decrease in the proliferation of the melanoma cells. Hence, a potential explanation for the elevated tumor progression seen in Mcpt6^−/−^ versus WT animals in vivo could be that Mcpt6 limits tumor cell proliferation. However, we cannot exclude that other mechanisms contribute to the effects of Mcpt6 deficiency on tumor progression in vivo. Of note, MCs express a variety of compounds of both potentially pro‐tumorigenic and anti‐tumorigenic nature (Marichal et al., 2013; Oldford & Marshall, 2015; Ribatti & Crivellato, 2009; Varricchi et al., 2017). We may thus envision that the exact expression profile of MCs present in a given tumor setting may determine their net impact on tumor growth. On a speculative angle, it is plausible that tumor‐associated MCs may exhibit polarization into pro‐tumorigenic (e.g., expressing high levels of pro‐tumorigenic growth factors such as VEGF) and anti‐tumorigenic subtypes (e.g., expressing high levels of tryptase), that is, in analogy with the polarization seen in the macrophage niche. However, further investigations are needed to evaluate this hypothesis.

## CONFLICT OF INTEREST

The authors declare no conflict of interest in relation to this work.

## AUTHOR CONTRIBUTIONS

Mirjana Grujic planned the study, performed most of the experimental work, interpreted data, and wrote parts of the manuscript; Fabio Rabelo Melo performed experiments and contributed to the writing of the manuscript; Ann‐Marie Gustafson performed experiments; Srinivas Akula expressed recombinant Mcpt6; Lars Hellman supervised the expression of Mcpt6 and gave input on the manuscript; and GP planned the study, interpreted the data, and wrote the manuscript.
