# Supplementary material for: Protective role of mouse mast cell tryptase Mcpt6 in melanoma
Source: Pigment Cell Melanoma Res. 2020 Jan 19;33(4):579–90. doi: 10.1111/pcmr.12859 (PMC7317424; doi:10.1111/pcmr.12859)
Supplement: Supplementary file 1 [file PCMR-33-579-s001.docx]

Suppl. Table 1. Primers used for qPCR analysis

| **Target** | **Forward primer (5`-3`)** | **Reverse primer (5`-3`)** |
| --- | --- | --- |
| GAPDH | CTC CCA CTC TTC CAC CTT CG | CCA CCA CCC TGT TGC TGT AG |
| mGbp10 | CTA ACC GGA AGT GTT TTG TC | CAG AAT CCC TAG TTT ATT CCC |
| Tgtp2 | GAG CAT TAG CCA CCA TTC CA | CAT CAT CCA GCC CAA AGT AAG A |
| Cxcl9 | GTT CGA GGA ACC CTA GTG ATA AG | GTT TGA GGT CTT TGA GGG ATT TG |
| Igtp | TGT CAC CGC CTT ACC AAT ATC | CAT CAG CCC GTG GTC TAA AT |
| F4/80 | AGG GTA TCA TGA GTT GAT GGC A | TGG AGC TTC ATA GTT GTA AGG CA |
| CD206 | GAG GGA AGC GAG AGA TTA TGG A | GCC TGA TGC CAG GTT AAA GCA |
| CD11c | GTG ACC CCG ATC ACT CTT CG | TCT GTC CAT AAG AGG CCG TG |
| CD8b1 | GAC GAA GCT GAC TGT GGT TGA | GCA GGC TGA GGG TGG TAA G |
| CD4 | AGG TGA TGG GAC CTA CCT CTC | GGG GCC ACC ACT TGA ACT AC |
| CPA3 | TGA CAG GGA GAA GGT ATT CCG | CCA AGG TTG ACT GGA TGG TCT |
| 5S rRNA | cat.# YP00203906* |  |
| mmu-miR-3098-5p | cat.# YP02105271 |  |
| mmu-miR-3098-3p | cat.# YP02119076 |  |
| mmu-miR-669b-5p | cat.# YP00205628 |  |
| mmu-miR-669b-3p | cat.# YP02119203 |  |

* reference for miRCURY LNA miRNA PCR Assays (Qiagen)
