# Supplementary material for: Protective role of mouse mast cell tryptase Mcpt6 in melanoma
Source: Pigment Cell Melanoma Res. 2020 Jan 19;33(4):579–90. doi: 10.1111/pcmr.12859 (PMC7317424; doi:10.1111/pcmr.12859)
Supplement: Supplementary file 2 [file PCMR-33-579-s002.docx]

**Suppl. Table 2.** Downregulated genes in tumors from Mcpt6^-/-^ vs. WT mice.

| **Probe.Set_ID** | **Gene name** | **Gene Description** | **Log2-fold change** |
| --- | --- | --- | --- |
| TC0300000849.mm.1 | Tchh | trichohyalin | -3,449 |
| TC1200002598.mm.1 | Ighv1-12 | immunoglobulin heavy variable V1-12 [Source:MGI Symbol;Acc:MGI:3646284] | -3,363 |
| TC1600002036.mm.1 | Krtap7-1 | keratin associated protein 7-1 | -3,239 |
| TC1100002649.mm.1 | Tgtp2 | T cell specific GTPase 2 | -3,053 |
| TC1500002270.mm.1 | Krt71 | keratin 71 | -2,746 |
| TC0500002755.mm.1 | Cxcl9 | chemokine (C-X-C motif) ligand 9 | -2,613 |
| TC0500002895.mm.1 | Gbp10 | guanylate-binding protein 10 | -2,573 |
| TC1600002024.mm.1 | Krtap22-2 | keratin associated protein 22-2 | -2,514 |
| TC1100002643.mm.1 | Gm12185 | predicted gene 12185 | -2,487 |
| TC1200002632.mm.1 | Ighv1-55 | immunoglobulin heavy variable 1-55 | -2,464 |
| TC1600000967.mm.1 | Gm10229 | predicted gene 10229 | -2,447 |
| TC1100004265.mm.1 | Igtp | interferon gamma induced GTPase | -2,357 |
| TC1600002025.mm.1 | Gm10228 | predicted gene 10228 | -2,350 |
| TC1100003716.mm.1 | Krtap1-5 | keratin associated protein 1-5 | -2,333 |
| TC1200002624.mm.1 | Ighv1-42 | immunoglobulin heavy variable V1-42 | -2,296 |
| TC1200002630.mm.1 | Ighv1-53 | immunoglobulin heavy variable 1-53 | -2,288 |
| TC1600002026.mm.1 | Krtap6-5 | keratin associated protein 6-5 | -2,2653 |
| TC1600000974.mm.1 | Gm7735 | predicted gene 7735 [Source:MGI Symbol;Acc:MGI:3649168] | -2,2650 |
| TC1600002035.mm.1 | Krtap8-1 | keratin associated protein 8-1 | -2,257 |
| TC0300001447.mm.1 | Gbp2 | guanylate binding protein 2 | -2,253 |
| TC0600003576.mm.1 | Igkv5-48 | immunoglobulin kappa variable 5-48 | -2,115 |
| TC1600002021.mm.1 | Krtap19-9a | keratin associated protein 19-9A [Source:MGI Symbol;Acc:MGI:3704466] | -2,099 |
| TC1600002020.mm.1 | Krtap19-5 | keratin associated protein 19-5 | -2,081 |
| TC0600003515.mm.1 | Igkv9-120 | immunoglobulin kappa chain variable 9-120 | -2,048 |
