# Supplementary material for: Protective role of mouse mast cell tryptase Mcpt6 in melanoma
Source: Pigment Cell Melanoma Res. 2020 Jan 19;33(4):579–90. doi: 10.1111/pcmr.12859 (PMC7317424; doi:10.1111/pcmr.12859)
Supplement: Supplementary file 3 [file PCMR-33-579-s003.docx]

| **Probe.Set_ID** | **Gene name** | **Gene Description** | **Log2-fold change** |
| --- | --- | --- | --- |
| TC0300001175.mm.1 | Mir669b | microRNA 669b | 3,482 |
| TC0200004648.mm.1 | Mir3098 | microRNA 3098 | 2,820 |
| TC0700001506.mm.1 | Gm19831 | PREDICTED: predicted gene, 19831, transcript variant 1 (Gm19831), miscRNA. | 2,482 |
| TC1100000339.mm.1 | Hba-a2 | hemoglobin alpha, adult chain 2 (Hba-a2), mRNA. | 2,235 |
| TC0700003908.mm.1 | Hbb-bt | hemoglobin, beta adult t chain | 2,112 |
| TC1100000336.mm.1 | Hba-a2 | hemoglobin alpha, adult chain 2 | 2,041 |
| TC0700003909.mm.1 | Hbb-bs | hemoglobin, beta adult s chain | 2,038 |
| TC1700001227.mm.1 | Gm11096 | predicted gene 11096 [Source:MGI Symbol;Acc:MGI:3779332] | 2,034 |
| TC1100002474.mm.1 | LOC100862145 | PREDICTED: uncharacterized LOC100862145, transcript variant 1 (LOC100862145), miscRNA. | 2,031 |
| TC0900003162.mm.1 | Mir5128 | microRNA 5128 | 2,016 |

**Suppl. Table 3.** Upregulated genes in the tumors from mMCP6^-/-^ mice vs. tumors from WT mice.
